# Time to share

**DOI:** 10.1186/1546-0096-11-5

**Published:** 2013-02-15

**Authors:** Nico M Wulffraat, Bas Vastert

**Affiliations:** 1 Department of Pediatric Rheumatology, UMCU, Utrecht, Netherlands

## Abstract

In the following a brief commentary is given on a new European project that aims to provide the European countries with recommendations for the care of children and yound adults with rheumatic diseases. These recommendations will be based on surveys sent to PRINTO members and systematic literature reviews. Surveys on current local standard of care and best practice will be send to PRINTO members in EU member states. The success of this project largely largely depends on information provided by individual centers from our existing PRINTO and PReS networks. We would therefore like to ask your collaboration in completing and returning these surveys which will be circulated March April 2013.

## 

In the course of 2013 a new European project called SHARE will be launched involving the pediatric rheumatology networks in all European Union members states and candidate member states with the aim to describe what is needed for optimal care for children and young people with rheumatic disease. This will be done using surveys that are sent to individual centers and organizations throughout Europe. The success of the project as described below largely depends on the collaboration from pediatric rheumatologists, allied health care professionals, parents and patient organizations. For a detailed overview of the current delivery of clinical care services, it is important that there is a high response rate with completion of as much data as possible. With this in mind, we provide a brief description of aims and future collective benefits of the SHARE initiative.

## Pediatric rheumatology in Europe: dealing with rare diseases

In April 2012, the European Agency for Health and Consumers (EAHC) signed the contract for a new European initiative for children and young persons with rheumatic diseases. The project description is “Single Hub and Access point for paediatric Rheumatology in Europe (acronym SHARE, project number 2011 1202, Project Coordinator NM Wulffraat). A specific problem for rare diseases is that their low prevalence hinders sound and representative research. As a consequence there is a lack of evidence based guidelines for disease (and pain) management of Pediatric Rheumatic Diseases (PRD) and treatment is mostly based on anecdotal observations and experience accrued by clinicians [1]. Therefore PRD treatment differs substantially throughout Europe and even within a single country. The international organizations for pediatric rheumatologists in Europe and the US, (PReS and PRCSG) have acknowledged this problem and jointly produced recently several recommendations in Juvenile Idiopathic Arthritis [2,3]. In addition, EULAR (see http://www.eular.org) promotes efforts to produce recommendations [4].

There is thus a need for standardized diagnosis and management of PRD throughout Europe. PRDs include both pediatric rheumatic autoimmune diseases and auto-inflammatory diseases. We expect sizable differences between institutions as well as differences between West and East Europe. Although valuable work on PRD is emerging - and has been - performed by various initiatives on both a national and a European level there is a clear need for facilitating better access to information by combining the expertise, the knowledge and resources available in Europe for individual diseases in one single PRD initiative. The resulting information network would facilitate sharing knowledge and expertise which can facilitate research initiatives to significantly improve the desired healthcare delivery in PRD.

The European Union has recognized this problem and states in its European Charter of patients’ rights (http://ec.europa.eu/health/ph_overview/co_operation/mobility/docs/health_services_co108_en.pdf) that “Despite their differences, national health systems in European Union countries place the same rights of patients, consumers, users, family members, weak populations and ordinary people at risk. Despite solemn declarations on the European Social Model (the right to universal access to health care), several constraints call the reality of this right into question. As European citizens, we do not accept that rights can be affirmed in theory, but then denied in practice, because of financial limits. Financial constraints, however justified, cannot legitimize denying or compromising patients’ rights” [5].

## Project description

The main goals are to summarize the needs for uniform management of rare pediatric rheumatic diseases throughout Europe, to provide recommendations for management of these diseases in European countries on which optimal treatment is based, to update the existing website with interactive tools and updated patient information, to provide a proposal for state of art postgraduate education and training for health care professionals dealing with these diseases (see Table 1). In short the aim of this project is to define what we need in order to provide optimal care to children with PRD in EU member and candidate member states. As is custom in European projects, Work Packages (WP) were formed to address these aims. Work packages 1–3 deal with management, dissemination of results and evaluation and will not be discussed in this paper.

**Table 1:** AIMS of the European SHARE project

Identifying the specific needs for optimal care in PRD in each EU country in order to achieve minimum standards of care ( WP leader P. Dezolezalova, Prague)	The goal is to identify the specific needs for optimal care in PRD in each EU country. Optimal care includes diagnosis, management of disease and providing both drug- and non-drug therapies. The country specific needs should be identified within 18 months after the start of the project.
Identifying best practices and establishing minimal standards of care for the treatment of patients suffering from PRD (WP leader B Vastert, Utrecht)	After completing a systematic literature review on treatment of PRD, the available evidence will be graded by organizing consensus meetings. The best practices identified should provide for minimal standards of care in the treatment of PRD throughout Europe
Establishing platforms for the exchange of information, data and samples and for linking the existing networks and projects. Ensuring a better foothold of PRD patients in both research and treatment (WP leader N Ruperto, Genoa)	A portal within the PReS site will link with existing registers, projects, etc. Simultaneously we will harmonize the way in which information is stored by providing for best practices and patient information. We will provide patients with knowledge on their diseases, treatment and research, provide access to patient specific networks including a platform for patients to express their views and provide feedback from parents on this project (via inclusion of patients in this project).
Identifying best practices for obtaining ethical consent and for data and sample collection in PRD (WP leader J Kummerle-Descher, Tubingen)	Identify best practices for obtaining ethical consent, data and sample collection. These actions are aimed at enforce the exchange of these data and samples between European centres and should increase the availability of data to facilitate performing large clinical studies more effectively.
Ensuring implementation of the best practices for training programmes on PRD healthcare professionals (WP leader A Martini, Genoa)	The goal is to disseminate up-to-date knowledge on PRD to healthcare professionals by providing a proposal for state of art postgraduate education. This proposal should be available at the end of the project.

Legend: *PRD*, Pediatric Rheumatic Diseases; *WP*, Work Package.

Defining the need for optimal care of PRD is the aim of a WP4, led by Pavla Dolazalova (Prague, Czech Republic). This WP will provide recommendations based on a detailed evaluation of current standards of care, access to care, and protocols. A good example of this is the recently published BSPAR (British Society for Paediatric and Adolescent Rheumatology) guidelines for the UK [6]. This overview will be based on the results of a survey that will be send to all pediatric rheumatology centers, including (but not limited to) the members of PReS and PRINTO networks. The survey will include all major PRDs. Question listed in this survey were provided by the members of the existing PReS working of the groups for JIA, SLE, JDM, Vasculitis and Periodic Fevers who are involved in the Share project.

To obtain a balanced representation from all parts of Europe we invited members of the existing PRES network to participate in the project (see list of participants of the SHARE consortium). Especially in countries with relatively few PReS members (such as in Eastern European countries) we will seek participation by additional centers providing care to children with PRD, such as adult rheumatological clinics.

WP5 (Bas Vastert, Utrecht, Netherlands) will address the issue of identifying best practices for treatment of paediatric patients suffering from PRDs: the activities within this goal start with a literature overview (as described in the EULAR method for achieving recommendations Guidelines on treatment of patients) by groups of experts from the PReS working groups [7]. After completing a systematic literature review, the available evidence will be graded, by organizing consensus meetings. Using the Delphi method consensus will be achieved on the minimum standards of care in PRD treatment.

WP6 (Nicola Ruperto, Genoa, Italy) will update a central platform for data collection and analysis and for sharing of information both for health care professionals and patients. The PRINTO and PReS are the largest European networks on PRDs (see http://www.PRES.org.uk and http://www.PRINTO.it). Activities within this WP are building further on an earlier EU SANCO grant with which patient information on PRD was documented and translated into all EU languages [8]. The PRINTO website providing that information is very succesfull, with around 11000 visitors per day. The PReS and PRINTO websites (http://www.pres.org.uk) is currently lacking sections on best practices, standards of care, interactive data comparison, and collaborative research projects. The idea is to strongly improve the website and to establish a network for data collection and analysis, to link existing networks such as the existing national JIA registers, and to provide tools for interactive use of this section that will enable benchmarking of local practices. The objective of providing a better foothold of patients in both research and treatment will be achieved by inclusion of patients and parents in the expert groups so that patients have direct influence on the results of the project. At the same time we want to improve a patients’ access to information and the ties between patients and their healthcare providers. This will be achieved by introducing better ways to disseminate the content through specific Google related techniques and social media that will make the website more visible including patient outreach in partnership with EURORDIS through its RareConnect (http://www.rareconnect.org) portal as well as its digital and social media presence.

A specific WP is devoted to making an inventory of national legal guidance within the EU (candidate) member states. Our experience is that the existing differences can impair international clinical research involving children [9]. The WP7 (Jasmin Kummerle-Deschner, Tübingen, Germany) will identify best practices for obtaining ethical consent from parents, children and adolescents, and for data and sample collection. To achieve this goal an analysis of ethical and legal issues surrounding data collection and procedures for informed consent will be performed. We will address our questions to national EU competent health authorities, local medical ethical committees and health care professionals. The results will be discussed in expert meetings in order to establish best practices for data exchange, the storage and shipment of DNA and viable cells.

Training and education is addressed in WP8 (Alberto Martini, Genoa, Italy). The aim here is to ensure the implementation of the best practices identified by the project by inclusion of the project’s results in training programmes. Based on a survey for existing national and international training programmes, a proposal will be drawn up for inclusion of the results in the existing training programme of PReS and its members. This proposal will be discussed with experts in a plenary session. The initiative will closely interact with the EULAR programme on accredited online educational courses (see http://www.eular.org), which will be a major activity for the PRES council in the next years [10].

## Time lines of the surveys in this project

Invitations to all PRINTO centers to complete this survey on current standards of care will be send early april 2013. We ask the respondents to complete their responses before july first, 2013. Results will be communicated early 2014 and during the annual PRES meetings. The project funding ends September 2015.

In summary, the SHARE initiative will circulate surveys to centers and organisations involved in providing care for children with PRD. Such surveys will form the basis of a thorough inventory of current local practice. From these a set of international best practices will be selected by. These practices will be presented to stakeholders such as health authorities, individual centers for pediatric rheumatic diseases, health care insurance companies and patient/parent organisations. The success of this project largely largely depends on information provided by individual centers from our existing PRINTO and PReS networks.

## Authors’ information

The SHARE consortium consists of the following partners (alphabetical order): Jordi Anton (Spain), Tadej Avcin (Slovenia), Brigitte Bader-Meunier (France), Michael Beresford (UK), Paul Brogan (UK), Liza McCann (UK), Tamas Constantin (Hungary), Dennis Costello (Eurordis), Jasmin Kummerle-Deschner(Germany), Pavla Dolezalova (Czech Republic), Gregor Dueckers (Germany), Ivan Foeldvari (Germany), Helen Foster (UK), Marco Gattorno (Italy), Marisa Giunta D’Albani (JIA Patient Organization, the Netherlands), Claudia Grave (JIA Patient Organization, Germany), Noortje Groot (the Netherlands), Veronique Hentgen (France), Gerd Horneff (Germany), Sylvia Kamphuis (the Netherlands), Isabelle Kone-Paut (France), Pekka Lahdenne (Finland), Bo Magnussen (Sweden), Alberto Martini (Italy), Kirsten Minden (Germany), JJ vd Net (the Netherlands), Seza Ozen (Turkey), Matthew Peak (UK), Clarissa Pilkington (UK), Joost Frenkel (the Netherlands), Bas Vastert (the Netherlands), Carine Wouters (Belgium), Nico Wulffraat (the Netherlands), Pierre Quartier (France), Angelo Ravelli (Italy), Annet v Royen (the Netherlands), Ingrida Rumba (Latvia), Nicola Ruperto (Italy), Silvia Scala (Italy) and Francesco Zulian (Italy).
